# Biomarker Effects in *Carassius auratus* Exposure to Ofloxacin, Sulfamethoxazole and Ibuprofen

**DOI:** 10.3390/ijerph16091628

**Published:** 2019-05-09

**Authors:** Xiaofan Yang, Xiaoping Xu, Xueyu Wei, Jie Wan, Yu Zhang

**Affiliations:** College of Biological and Chemical Engineering, Anhui Polytechnic University, Wuhu 24100, China; xiaofan108@ahpu.edu.cn (X.Y.); wxyu1027@126.com (X.W.); nbz022@126.com (Y.Z.)

**Keywords:** biomarker, *Carassius auratus*, environmentally relevant concentration, pharmaceutical

## Abstract

Ofloxacin, sulfamethoxazole and ibuprofen are three commonly used drugs which can be detected in aquatic environments. To assess their ecotoxicity, the effects of these three pharmaceuticals and their mixture on AChE (acetylcholinesterase) activity in the brain, and EROD (7-ethoxyresorufin-O-deethylase) and SOD (superoxide dismutase) activities in the liver of the freshwater crucian carp *Carassius auratus* were tested after exposure for 1, 2, 4 and 7 days. The results showed that treatments with 0.002–0.01 mg/L ofloxacin and 0.0008–0.004 mg/L sulfamethoxazole did not significantly change AChE, EROD and SOD activities. AChE activity was significantly inhibited in response to treatment with >0.05mg/L ofloxacin and >0.02 mg/L sulfamethoxazole. All three biomarkers were induced significantly in treatments with ibuprofen and the mixture of the three pharmaceuticals at all the tested concentrations. The combined effects of ofloxacin, sulfamethoxazole and ibuprofen were compared with their isolated effects on the three biomarkers, and the results indicated that exposure to ibuprofen and the mixture at environmentally relevant concentrations could trigger adverse impacts on *Carassius auratus*. The hazard quotient (HQ) index also demonstrated a high risk for ibuprofen. Moreover, the present study showed that the effects of ofloxacin, sulfamethoxazole and ibuprofen might be additive on the physiological indices of *Carassius auratus*.

## 1. Introduction

Pharmaceuticals and personal care products (PPCPs) include thousands of chemicals, such as antibiotics, non-steroidal anti-inflammatory drugs, blood lipid lowering agents, analgesics and β-blockers, detergents, and cosmetics [1]. Due to anthropogenic activities, a large quantity of pharmaceutical contaminants has been discharged into aquatic systems [2,3]. The fate and toxicity of PPCPs in environments are attracting increasing concern, and some drugs can exert physiological stress on aquatic organisms in the wild or under experimental exposure [4]. Frequently used pharmaceuticals, such as sulfamethoxazole, ofloxacin and ibuprofen, can now be detected in surface water [5,6].

Sulfamethoxazole is a typical sulfonamide antibiotic, which is widely used for the treatment of tonsillitis, acute bronchitis, pulmonary infection, urinary tract infections, and veterinary use in aquaculture practices [7]. Ofloxacin is a synthetic chemotherapeutic antibiotic of fluoroquinolone, which is the most commonly used to treat infections in medicine [8,9] and aquaculture [10]. Ibuprofen is one of the most salable non-steroidal anti-inflammatory drugs, which are used to relieve symptoms of arthritis, rheumatic disorders, pain, and fever [11]. Sulfamethoxazole, ofloxacin and ibuprofen are frequently used in practice but cannot be removed effectively by conventional wastewater treatments [12,13]. The continuous discharge of these drugs could lead to a persistent threat to aquatic organisms.

Ofloxacin at 200 μg/L can cause a significant decrease in the number of offspring at first reproduction in F3 (third generation of clone) and F4 of *Daphnia magna*. Sulfamethoxazole did not exhibit obvious effects on the endpoints of reproduction and growth at 80.0 μg/L. For ibuprofen, the age at first reproduction was delayed significantly in F3 and F4 and the offspring number decreased obviously in F3, F4, and F5 at 900.0 μg/L [16]. These data solely assessed the effects of these compounds on aquatic organisms. In reality, the permanent release of these three compounds into aquatic environments should result in their coexistence in surface water, which was generally detected at the level of ng/L or μg/L [2,4,5,6] (Table 1). Although these concentrations were lower than the effective level on *Daphnia magna* in our previous research [16], whether the combined pollution of these compounds had effects on aquatic organisms remains unknown. These pieces of information should be important to evaluate the toxicity of ofloxacin, sulfamethoxazole and ibuprofen in real environments.

Ofloxacin, sulfamethoxazole and ibuprofen are not as highly toxic as pesticides or persistent organic pollutants, however, continuous discharge could lead to false persistence and a threat to aquatic organisms. Fish are beings that are untargeted by most pharmaceuticals, and information is still lacking on the physiological effects upon crucian carp in vivo of exposure to ofloxacin, sulfamethoxazole and ibuprofen, especially at environmentally relevant concentrations. *Carassius auratus* is a commonly used organism for toxicity assessments and acetylcholinesterase (AChE), 7-ethoxyresorufin-O-deethylase (EROD) and superoxide dismutase (SOD) activities in *Carassius auratus* are sensitive biomarkers for describing the toxic effects of pollutants on aquatic organisms [17,18,19]. In the present study, to investigate the combined effects of ofloxacin, sulfamethoxazole and ibuprofen on the physiology of *Carassius auratus*, changes in AChE, EROD and SOD activities were tested in response to treatments with these three compounds individuallyor in combination. These data could be useful for accurately evaluating the environmental hazards of these pharmaceuticals. Also, these data could provide basic information to assess the possibility of applying these biomarkers as indicators of the aquatic contamination of pharmaceuticals.

## 2. Materials and Methods

### 2.1. Chemicals

Ofloxacin, sulfamethoxazole and ibuprofen were purchased from Japan Wako Pure Chemical Industries, Ltd., and the purities were all above 98%. Acetylthiocholine iodide (ATChI), β-nicotinamide adenine dinucleotide 2′-phosphate reduced tetrasodium salt (NADPH), 1-chloro-2, 4-dinitro-benzene (CDNB) and glutathione reductase (GR) were obtained from Sigma Chemical Company (St. Louis, MO, USA). Coomassie brilliant blue G-250 (Ultra-Pure Grade) and 5, 5′-dithiobis (2-nitrobenzoic acid) (DTNB) were purchased from Sinopharm Chemical Reagent Co., Ltd. (Shanghai, China). All other chemicals were of analytical grade and were obtained from Shanghai Chemical Reagent Co., Ltd. (Shanghai, China).

### 2.2. Test Organisms

The use of fish for research purposes in this study followed ethical guidelines. *Carassius auratus* (14 ± 2 g), which had not been exposed to any pharmaceuticals, were obtained from the Nanjing Institute of Fishery Science. The fish were acclimatized for at least 2 weeks in dechlorinated water at 17 to 18 °C, with pH of 7.2 ± 0.2, dissolved oxygen of 5.8 ± 0.2 mg/L, and a natural photoperiod of 11 h light and 13 h dark.

### 2.3. Exposure Test

Prior to the experiment, fish were starved for 24 h. The pharmaceuticals were first dissolved in DMSO (dimethyl sulfoxide) and further diluted using dechlorinated tap water. Five concentrations each were prepared for ofloxacin (0.002, 0.01, 0.05, 0.25 and 1.25 mg/L), sulfamethoxazole (0.0008, 0.004, 0.02, 0.1 and 0.5 mg/L), ibuprofen (0.009, 0.045, 0.225, 1.125, 5.625 mg/L) and their mixture (M1-M5). For combined exposure, the three chemicals were mixed together in order of concentration. For example, M1 contained 0.002 mg/L ofloxacin, 0.0008 mg/L sulfamethoxazole and 0.009 mg/L ibuprofen. At the same time, a blank control and a solvent control with 0.05% DMSO were also prepared. During the experiments, no food was provided and the experimental solution was renewed every day. Water temperature, pH value, dissolved oxygen and natural photoperiod were the same as those during the adaptation period. Fish were randomly assigned into different groups. For each group, 12 fish were raised in 30 L glass tanks (40 × 25 × 30 cm) containing 20 L of experimental solution under constant aeration. Three fish were sacrificed from each treatment at 1, 2, 4 and 7 days of exposure, and the brain and liver tissues were collected immediately for enzyme assays.

### 2.4. Chemical Analysis

Water samples were collected from each glass tank 1, 2, 4 and 7 days after the exposure. The concentration of each chemical was analyzed using an Agilent 1260 UHPLC (Agilent Technologies, Santa Clara, CA, USA) instrument equipped with a 6460QQQ triple quadrupole mass spectrometer with electrospray ionization. Positive ion mode was used for sulfamethoxazole and ofloxacin, while negative ion mode was used for ibuprofen. Quantification of chemicals was performed using the external standard method. The extraction recoveries of the analytes were estimated using ultrapure water spiked with the analytes at a concentration of 10 μg/L. The precision of the method was determined by the repeated analysis of standards (*n* = 3) of a blank water sample. The method was considered accurate if recoveries were 70–120%, and precision was satisfactory if the relative standard deviation (RSD) was lower than 15%. Recoveries, RSD and detection limits (DL) of the method are given in Table 2.

### 2.5. Enzyme Assay

Three individuals were sampled from each treatment at day 1, 2, 4, and 7 after exposure. The brain and liver tissues were collected immediately, washed with 0.15 M KCl and then stored at −80 °C [20]. Brain samples were homogenized in nine volumes of cold phosphate buffer (0.1 M, pH 7.2, 0.1% Triton X-100) and centrifuged for 20 min at 10,000× *g* at 4 °C. The supernatants were collected to determine the activity of AChE using the method described by Guilhermino et al. [21]. The unit of AChE activity was defined as nmol/mg protein/min.

Liver samples were homogenized in nine volumes of cold buffer (0.25 M sucrose, 0.1 M Tris-HCl, 1 mM EDTA, pH 7.4) and centrifuged for 15 min at 10,000× *g* at 4 °C. EROD activity was monitored according to Lu et al. [22]. EROD activity was expressed as pmol/mg protein/min. SOD activity was determined by measuring the inhibition of the auto-oxidation of pyrogallol at 420 nm [23]. SOD activities were expressed as U/mg protein. One unit of SOD activity was defined as the enzyme causing 50% inhibition of pyrogallol auto-oxidation. Protein concentrations were determined by Bradford’s method (1976), with bovine serum albumin as the standard.

### 2.6. Statistical Analysis

The results for each biomarker are presented as mean ± SD (*n* = 3). The effects of pharmaceuticals on each biomarker were analyzed by one-way analysis of variance (ANOVA) and statistically different treatments were identified by Dunnett’s test. All differences were considered significant at *p* < 0.05. Statistical analyses were performed using the SPSS statistical package (ver. 12.0, SPSS Company, Chicago, IL, USA).

### 2.7. Calculation of Hazard Quotient

Hazard quotient (HQ) has been confirmed as an effective method to interpret biomonitoring results [24,25]. In this study, HQ was applied to assess the potential ecotoxicological risk of pharmaceutical contaminants to *Carassius auratus*. HQ value was estimated as the ratio of MEC (measured environmental concentration)/PNEC (predicted non-effect concentration). The MEC was the average of the measured concentrations. NOEC (no observed effect concentration) of ofloxacin, sulfamethoxazole and ibuprofen was calculated based on the data analysis of the three biomarkers in the present study. AF (assessment factor) 10 was chosen for ofloxacin, sulfamethoxazole, and ibuprofen in order to ensure ecological safety. PNEC was the ratio of NOEC/AF.

## 3. Results and Discussion

### 3.1. Verification of Exposure Concentration

Before the exposure period, the selected pharmaceuticals were measured and thos data are given in Table 2. The measured concentrations were in the range of 84.3–120.5%, 87.5–117.5% and 84.7–96.4%, compared with the nominal values for ofloxacin, sulfamethoxazole and ibuprofen, respectively. Since a good agreement was exhibited between the nominal and measured exposure concentrations, the subsequent analyses of biological effects were based on the nominal concentrations.

### 3.2. AChE Activity

No mortality occurred during the experiments. AChE is an enzyme that catalyzes the hydrolysis of acetylcholine into choline and acetate in the synaptic cleft. When AChE is inhibited, the neurotransmitter acetylcholine (ACh) cannot be hydrolyzed in nerve synapses and neuromuscular junctions, causing an abnormal amount of ACh at these sites. These changes lead to overactivation of brain and muscular tissues [26]. If the activity of AChE decreases, neuronal and muscular injury may occur. As previously reported, in response to pollutants, AChE activity was inhibited in fish, which subsequently affected their growth, survival, feeding and reproductive behaviors [27,28]. In most studies regarding the toxicity of pharmaceuticals, the activity of AChE was inhibited in fish, such as in studies of organophosphates and carbamates [29,30]. However, a few inductive effects of AChE were reported in fish when exposed to contaminants. For example, exposure to clofibric acid significantly increased AChE activity in the brains of mosquito fish (*Gambusia holbrooki*) [31].

**Figure 1:**
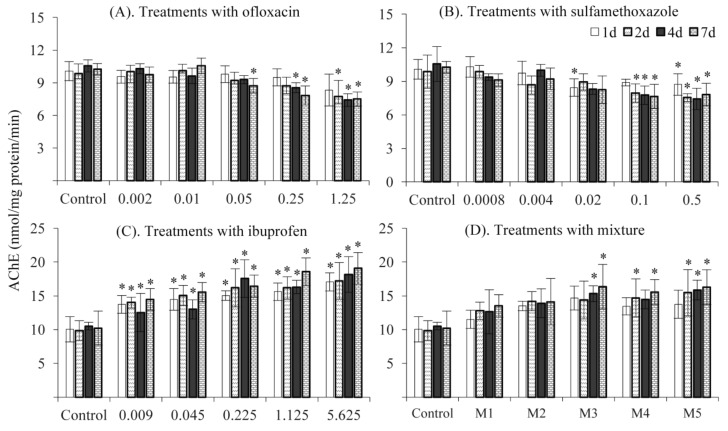
Acetylcholinesterase (AChE) activity in response to treatments with ofloxacin (**A**), sulfamethoxazole (**B**), ibuprofen (**C**) and their mixture (**D**). *: significantly different from the control.

In the present study, AChE activity was monitored at different timeslots (Figure 1). At environmentally relevant concentrations, sole treatments with ofloxacin (0.002 and 0.01 mg/L) and sulfamethoxazole (0.0008 and 0.004 mg/L) revealed no significant effects on AChE activity. However, treatments with 0.25 and 1.258 mg/L ofloxacin as well as 0.5 mg/L sulfamethoxazole significantly inhibited AChE activity at day 4 or 7 (Figure 1A,B). These results partially agree with Li et al. [29] who revealed that treatment with 50 mg/L sulfamethoxazole decreased AChE activity by 30% in goldfish and suggested that high levels of ofloxacin and sulfmethoxazole might negatively affect fish in environments. In contrast, treatments with ibuprofen and the mixture of the three pharmaceuticals increased the AChE activity (Figure 1C). The biological explanation for the AChE induction was unclear. Perhaps fish were able to compensate for injurious toxicological stresses by enhancing AChE activity [27]. Moreover, the treatment with the mixture of the three pharmaceuticals also increased the AChE activity (Figure 1D), but the level of AChE activity was slightly lower than that with ibuprofen, suggesting that the inductive effects of ibuprofen could be partially neutralized by sulfamethoxazole and ofloxacin. At the same concentration, there were no significant differences among treatments at different timeslots, which might reveal that these three pharmaceuticals did not accumulate in fish.

### 3.3. EROD Activity

EROD is one of the hepatic mixed-function oxidase enzymes and is considered as a common indicator of many xenobiotics. EROD activity is a monitoring tool for cytochrome P4501A (CYP1A) inducers [32], and has been widely used as a biomarker to assess the effects on fish of chemicals which can bind to the aryl hydrocarbon receptor (e.g., polycyclic aromatic hydrocarbons (PAHs), polychlorinated biphenyls (PCBs), dioxins, and dibenzofurans) [33]. In response to toxic chemicals, EROD is generally induced to upregulate the antioxidant functions. For example, Martín-Díaz et al. [34] found that exposure to 0.4 mM methotrexate significantly induced EROD activity in the mussel (*Elliptio complanata*). The EROD activity in the gills and liver of the rainbow trout (*Oncorhynchus mykiss*) increased by 28 to 58 times after exposure to 200 µg/L of propranolol [35]. However, excessive levels of pharmaceuticals lead to a decrease in enzyme activity. For instance, Laville et al. [36] found that treatments with low concentrations of propranolol unregulated EROD activity, while high concentrations inhibited EROD activity. The enzyme in the organism was damaged at excessive exposure levels and, consequently, the enzyme activity decreased.

**Figure 2:**
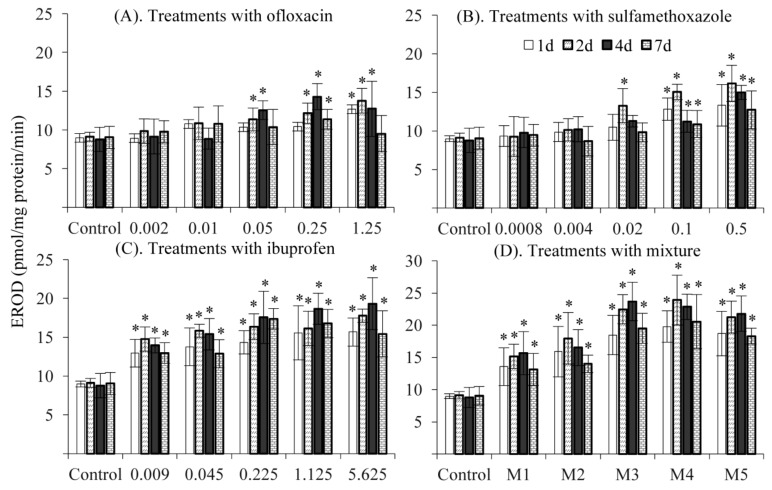
7-ethoxyresorufin-O-deethylase (EROD) activity in response to treatments with ofloxacin (**A**), sulfamethoxazole (**B**), ibuprofen (**C**) and their mixture (**D**). *: significantly different from the control.

In the present study, treatments with environmentally relevant concentrations of ofloxacin (0.002 and 0.01 mg/L) and sulfamethoxazole (0.0008 and 0.004 mg/L) did not change EROD activity during the exposure period (Figure 2A,B), whereas treatments with these two antibiotics significantly increased EROD activity at high concentrations. The maximum induction of EROD activity was observed at day 4 for ofloxacin and at day 2 for sulfamethoxazole. Treatments with ibuprofen and the mixture significantly increased EROD activity at all tested concentrations and at all timeslots (Figure 2C,D). The inductive effects of the mixtures on EROD activity were similar to those in treatments with ibuprofen, but the level of EROD activity in treatments with the mixture was higher in comparison to treatments with ibuprofen. These results suggest that additive effects exist among treatments with ofloxacin, sulfamethoxazole and ibuprofen.

### 3.4. SOD Activity

In order to resist oxidation, cells increase their SOD expression levels since SOD can dismutate superoxide radicals to form hydrogen peroxide (H_2_O_2_) and oxygen (O_2_) [37], which forms the first line of defense against superoxide radicals. Their inducibility in response to oxidative stress is an important feature of SOD and can be used as an indicator of environmental stresses [38]. Li et al. found that the activities of SOD in the brains of juvenile rainbow trout were induced when exposed to 0.27 mg/L verapamil after a short-term period [39], and hepatic SOD activities significantly increased after exposure to 200 μg/L carbamazepine for 21 and 42 days [40]. The activation of SOD counterbalances the production of free radical intermediates via transforming them into oxygen and hydrogen peroxide.

**Figure 3:**
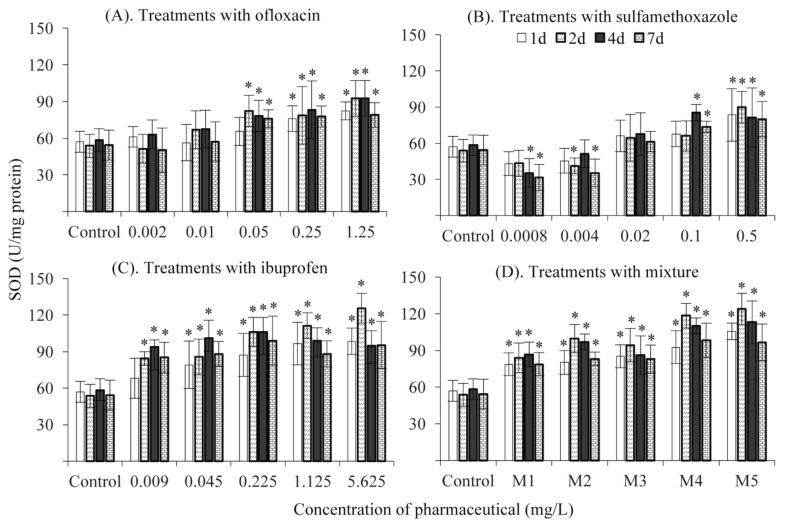
Superoxide dismutase (SOD) activity after exposure to ofloxacin (**A**), sulfamethoxazole (**B**), ibuprofen (**C**) and their mixture (**D**). *: significantly different from the control.

In the present study, treatment with ≥0.05 mg/L ofloxacin significantly increased SOD activity, but no effects were observed in treatments at environmentally relevant concentrations (Figure 3A). Treatments with high concentrations of sulfamethoxazole (≥0.1 mg/L) also significantly increased SOD activity. Nevertheless, SOD activity was significantly suppressed in treatments with the two lowest concentrations of sulfamethoxazole at days 4 and 7 (Figure 3B). The decrease in SOD activity at low concentrations of sulfamethoxazole seemed unexpected. However, this was not an isolated event. Previously, Binelli et al. [40] revealed a similar pattern of SOD activity variation during an investigation into the effects of antimicrobial trimethoprim on the freshwater mussel *D. polymorpha*. This phenomenon might be due to the reduced bacterial charge in fish. In general, lots of bacteria exist in the bodies of fish, which may produce poisonous secretions and subsequently trigger superoxidation. Most of these bacteria are sensitive to sulfamethoxazole [41,42]. Exposure to a low level of sulfamethoxazole should inhibit the growth of bacteria and consequently decrease the baseline level of antioxidant enzymes. The uncertain responses of SOD activity made it more complex to adopt SOD as a biomarker of antibiotic contaminations in fish.

Treatments with ibuprofen and the mixture significantly increased SOD activity at all tested concentrations, compared with the control, which was consistent with Parolini et al. [11] who found that SOD activity increased in the freshwater mussel *D. polymorpha* after exposure to 0.2 μg/L of ibuprofen. A positive relationship was observed between SOD activity and the concentration of chemicals (Figure 3C,D). The change pattern of SOD activity in treatments with the mixture was almost the same as those with ibuprofen along with the increasing concentration and elongated time, suggesting that ibuprofen, rather than sulfamethoxazole and ofloxacin, might be the effective chemical in the mixture.

### 3.5. Environmental Implications

Chemical analysis of pharmaceutical contaminants in aquatic environments is of limited use unless these data are related to their potential ecological effects via a risk assessment approach. The HQ values were assessed by comparing with a commonly used risk screening benchmark for ecotoxicological effects from contaminated water [43,44]. In the present study, hazard quotient (HQ) was applied to assess the potential ecotoxicological risk of pharmaceutical contaminants to *Carassius auratus* (Table 3). HQ value was estimated as the ratio of MEC (measured environmental concentration)/PNEC (predicted non-effect concentration). The MEC was the average of the measured concentrations in Table 1. NOEC of ofloxacin, sulfamethoxazole and ibuprofen was calculated based on the data analysis of the three biomarkers in the present study. AF (assessment factor) 10 was chosen for ofloxacin, sulfamethoxazole, and ibuprofen in order to ensure ecological safety. PNEC was the ratio of NOEC/AF.

Ofloxacin showed no significant risk (HQ < 1), with an average index of 0.755. Sulfamethoxazole showed a low significant risk (1.0 ≤ HQ < 10), with a high HQ index of 2.77. Additionally, ibuprofen demonstrated a significant potential adverse effect (10 ≤ HQ < 100), with the highest HQ index at 24.78. The HQ index of each compound is mainly based on its toxicological potency and detected concentration in the water body. To date, the ecotoxicological evaluation of chemical contamination is still difficult in aquatic environments.

Solely based on the laboratory tests, it is almost impossible to apply the HQ index in field assessment, because of the variety of pharmaceutical pollutants present in surface water. However, the additive effects of the three tested pharmaceuticals on the EROD activity probably implied a potential high risk in the filled polluted area.

## 4. Conclusions

In the present study, the effects of ofloxacin, sulfamethoxazole, ibuprofen and their mixture on AChE, EROD and SOD activities in *Carassius auratus* were determined. The results showed that treatments with ofloxacin and sulfamethoxazole at environmentally relevant concentrations did not significantly change AChE, EROD and SOD activities. In comparison, treatments with low concentrations of sulfamethoxazole significantly decreased SOD activity. Exposure to high concentrations of ofloxacin and sulfamethoxazole showed slight toxicity to *Carassius auratus*. Treatments with ibuprofen and the pharmaceutical mixture significantly increased EROD and SOD activities even at environmental concentrations, suggesting a high environmental risk for sulfamethoxazole. Combined exposure to the mixture of the three pharmaceuticals showed an additive effect on EROD activity and an antagonistic effect on AChE activity, suggesting that more attention should be paid to the interactions among pharmaceuticals during the assessment of their toxicity.

## Figures and Tables

**Table 1:** Basic information about ofloxacin, sulfamethoxazole, and ibuprofen, and co-detection concentrations in surface water.

Pharmaceutical Information	Environmentally Measured Concentration (μg/L)
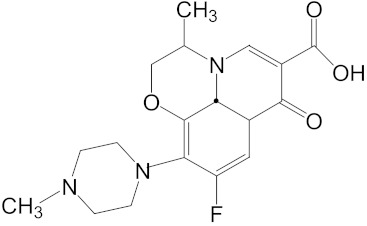 Ofloxacin (Molecular Weight= 361.38, logKow = −0.39)	0.11, Shenzhen River [6] 8.77, Llobregat River [5] 0.18, Haihe River [14]
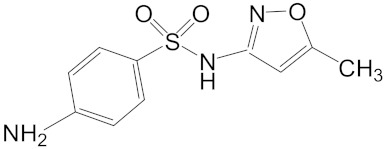 Sulfamethoxazole (Molecular Weight = 253.3 logKow = 0.89)	0.88, Shenzhen River [6] 11.92, Llobregat River [5] 0.5, England Stream [4]
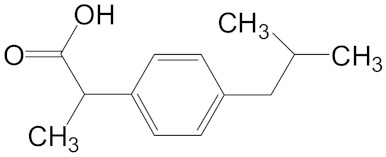 Ibuprofen (Molecular Weight = 206.29, logKow = 3.97)	9.89, Llobregat River [5] 1.11, England Stream [4] 2.37, Tyner River [15]

**Table 2:** The nominal and measured concentrations (MC) of the selected pharmaceuticals and the quality assurance of the chemical analysis.

Pharmaceutical	Nominal Concentration (NC) & Measured Concentration (MC)						Recovery Rate (%)	RSD (%)	DL (ng/L)
Ofloxacin	NC	0.002	0.01	0.05	0.25	1.25	100.7	6.2	2
	MC	0.002	0.012	0.046	0.21	1.25			
Sulfamethoxazole	NC	0.0008	0.004	0.02	0.1	0.5	85.2	4.7	1
	MC	0.0007	0.0047	0.0213	0.1022	0.5121			
Ibuprofen	NC	0.009	0.045	0.225	1.125	5.625	83.6	4	2
	MC	0.084	0.041	0.212	0.953	5.421			
MIX	NC	0.002 + 0.0008 + 0.009	0.01 + 0.004 + 0.045	0.05 + 0.02 + 1.125	0.25 + 0.1 + 1.125	1.25 + 0.5 + 5.625			
	MC	0.002 + 0.0008 + 0.009	0.013 + 0.0036 + 0.045	0.056 + 0.022 + 0.243	0.264 + 0.0.119 + 0.017	1.119 + 0.486 + 5.501			

Recovery rate: the percentage value of nominal concentration and measured concentration; RSD: relative standard deviation; DL: detection limit.

**Table 3:** The values of NOEC (no observed effect concentration), AF (assessment factor), PNEC (predicted non-effect concentration), MEC (measured effect concentration) and HQ (hazard quotient).

Pharmaceutical	NOEC (µg/L)	AF	PNEC (µg/L)	MEC (µg/L)	HQ Value
Ofloxacin	39.68	10	3.97	3.02	0.76
Sulfamethoxazole	16.02	10	1.60	4.43	2.77
Ibuprofen	1.80	10	0.18	4.46	24.78
